# Exploring the Pharmacological and Clinical Features of Lumateperone: A Promising Novel Antipsychotic

**DOI:** 10.3390/ijms252413289

**Published:** 2024-12-11

**Authors:** Magdalena Sowa-Kućma, Patrycja Pańczyszyn-Trzewik, Rafał R. Jaeschke

**Affiliations:** 1 Department of Human Physiology, Institute of Medical Sciences, Medical College of Rzeszów University, Kopisto 2a, 35-315 Rzeszów, Poland; 2 Centre for Innovative Research in Medical and Natural Sciences, Medical College of Rzeszów University, Warzywna 1a, 35-310 Rzeszów, Poland; 3 Section of Affective Disorders, Department of Psychiatry, Jagiellonian University Medical College, Kopernika 21a, 31-501 Kraków, Poland

**Keywords:** lumateperone, antipsychotic, schizophrenia, serotonin modulation, dopamine receptors, neuropsychiatric disorders

## Abstract

Lumateperone is a novel antipsychotic recently approved for the treatment of schizophrenia. Its unique pharmacological profile includes modulation of serotonergic, dopaminergic, and glutamatergic neurotransmission, differentiating it from other second-generation antipsychotics. This paper explores the pharmacological features and clinical potential of lumateperone across neuropsychiatric conditions. A review of current literature, including pharmacokinetic and pharmacodynamic studies, was conducted. It focused on lumateperone’s mechanism of action and receptor-binding profile, and clinical trials assessing its efficacy and safety in schizophrenia and other psychiatric disorders. Lumateperone demonstrates high affinity for 5-HT_2A_ receptors, moderate affinity for D_2_ receptors, and low affinity for H_1_ and 5-HT_2C_ receptors. It acts as a presynaptic D_2_ agonist and a postsynaptic antagonist, contributing to a favorable side-effect profile with reduced extrapyramidal symptoms. Clinical trials suggest that lumateperone is effective in reducing both positive and negative symptoms of schizophrenia, with minimal metabolic and cardiovascular risks. It is also being explored as an adjunctive therapy for major depressive disorder and bipolar depression. Lumateperone presents a promising therapeutic option for schizophrenia with a novel mechanism of action and a favorable safety profile. Its potential application in other psychiatric conditions warrants further investigation, particularly in treatment-resistant populations.

## 1. Introduction

Schizophrenia is a chronic and debilitating mental disorder affecting approximately 1% of the global population. Despite significant advancements in pharmacotherapy, many patients continue to experience inadequate symptom control, treatment resistance, or intolerable side effects associated with existing antipsychotic medications. Second-generation antipsychotics (SGAs) have improved the management of psychotic symptoms; however, their use is frequently accompanied by metabolic, cardiovascular, and extrapyramidal side effects. These adverse effects can significantly impact patient adherence to treatment and overall quality of life. As a result, there is a pressing need for novel antipsychotic agents that provide improved efficacy while minimizing safety concerns.

The introduction of lumateperone as a novel antipsychotic agent marks a significant advancement in the pharmacological landscape for the treatment of schizophrenia and potentially other neuropsychiatric disorders. Lumateperone’s distinctive mechanism of action engages with serotoninergic, dopaminergic, and glutamatergic systems, providing a multifaceted approach to neurotransmission. This stands in stark contrast to conventional antipsychotics, which predominantly target dopaminergic pathways and frequently result in a higher prevalence of extrapyramidal symptoms (EPS) [1,2].

Lumateperone is a recently introduced antipsychotic approved by the U.S. Food and Drug Administration (FDA) for the treatment of schizophrenia and bipolar depression in adults [3]. However, it is not currently approved for use in the European Union (EU). The manufacturer has submitted an application for regulatory approval in the EU, and evaluations are ongoing in select member states. Although the drug’s availability remains regionally restricted, understanding its unique pharmacological profile and therapeutic potential is globally relevant. Lumateperone represents a new generation of antipsychotics with distinctive mechanisms of action that hold promise for advancing psychiatric treatment strategies worldwide and informing future regulatory decisions.

The unique pharmacological profile of lumateperone involves simultaneous modulation of serotonergic, dopaminergic, and glutamatergic neurotransmission, setting it apart from other SGAs. Specifically, lumateperone acts as a potent antagonist at 5-HT_2A_ receptors, a moderate antagonist at postsynaptic D_2_ receptors, and a presynaptic partial agonist at D_2_ receptors. Additionally, it inhibits the serotonin transporter (SERT) and modulates glutamate neurotransmission through enhancement of N-methyl-D-aspartate receptor (NMDAR) function and upregulation of alpha-amino-3-hydroxy-5-methyl-4-isoxazolepropionic acid receptor (AMPAR) activity via the mammalian target of rapamycin (mTOR) pathway.

This multifaceted mechanism of action offers potential advantages in treating both the positive and negative symptoms of schizophrenia, as well as cognitive deficits and mood disturbances. Furthermore, its favorable side-effect profile, characterized by a lower risk of extrapyramidal symptoms and metabolic complications, positions lumateperone as a promising therapeutic option for patients with significant unmet needs.

Given the recent introduction of lumateperone into clinical practice and ongoing research into its broader therapeutic applications, a comprehensive review of its pharmacological and clinical features is both timely and clinically significant. This review aims to provide an updated and detailed analysis of lumateperone’s mechanisms of action, clinical efficacy, safety profile, and potential roles in treating various neuropsychiatric disorders beyond schizophrenia.

## 2. Pharmacology

Bristol Myers Squibb initially developed the drug, which is currently manufactured by Intra-Cellular Therapies under the development codes ITI-007 (lumateperone tosylate salt) and ITI-722 (lumateperone) [4]. On 20 December 2019, the US Food and Drug Administration (FDA) approved lumateperone, marketed under the brand name CAPLYTA^®^, for the treatment of schizophrenia in adults, following evidence from three double-blind, placebo-controlled trials (NCT01499563, NCT02282761, NCT02469155) [3,5]. The medication has been available as oral capsules since February 2020, recommended for once-daily intake with food, preferably before bedtime, without the need for dose titration [3,5]. Although clinical studies evaluated both lower (14 and 28 mg) and higher (84 mg) doses, the FDA approved only the 42 mg dose (equivalent to 60 mg of lumateperone tosylate) for this specific indication [3].

Further clinical trials are ongoing to explore additional uses of lumateperone, including its application in pediatric patients with schizophrenia or schizoaffective disorder, and for the prevention of relapse in patients with schizophrenia, bipolar depression, or major depressive disorder (MDD). It is also hypothesized that lumateperone may be an effective adjunctive treatment for patients with MDD [6].

### 2.1. Basic Pharmacology

**Figure 1:**
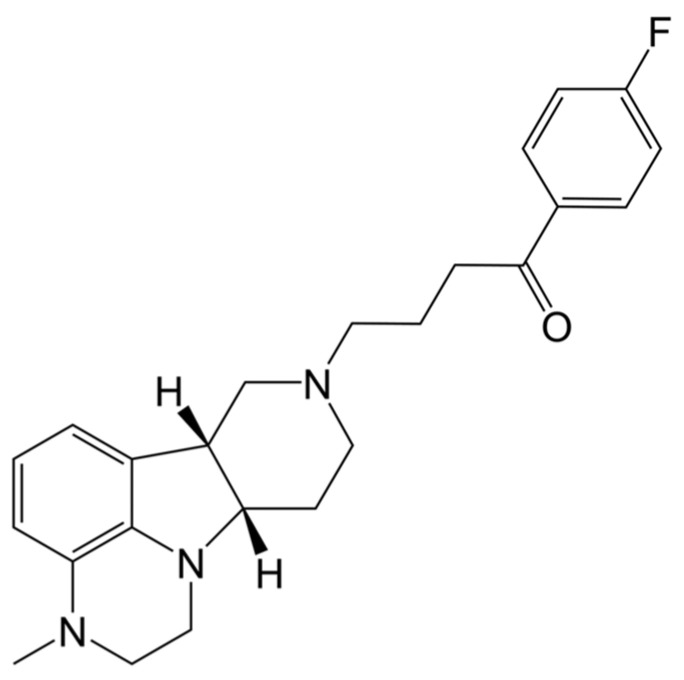
Chemical structure of lumateperone tosylate (created in BioRender).

CAPLYTA^®^ capsules contain lumateperone in the form of lumateperone tosylate salt (ITI-007), which is chemically named 1-(4-fluorophenyl)-4-(3-methyl-2,3,6b,9,10,10a-hexahydro-1H-pyrido [3′,4′:4,5]pyrrolo[1,2,3-e]quinoxalin-8(7H)-yl)butan-1-one 4-methylbenzenesulfonate. Lumateperone is a butyrophenone derivative, making it structurally related to haloperidol and droperidol (a first-generation typical antipsychotic; FGA) or melperone (an atypical antipsychotic). The molecular formula of lumateperone tosylate is C_31_H_36_FN_3_O4_S_, and its molecular weight is 565.7 g/mol. The structural formula is illustrated in Figure 1.

Lumateperone is a potent antagonist of the 5-HT_2A_ receptor and an inhibitor of the serotonin transporter (SERT) [7,8]. Additionally, the drug exhibits a location-dependent effect on D_2_ receptors: It functions as a partial agonist at presynaptic D_2_ receptors and as an antagonist at postsynaptic D_2_ receptors [9]. Furthermore, lumateperone acts as a dopamine phosphoprotein modulator. A D_1_ receptor-dependent mechanism indirectly influences glutamatergic signaling through phosphorylation of the GluN2B subunit of the NMDAR [10]. Moreover, lumateperone enhances NMDAR and AMPAR signaling in the prefrontal cortex, although the precise pharmacological mechanisms underlying this modulation remain unknown [11,12].

Lumateperone has a relatively low affinity for H_1_, 5-HT_2C_, and muscarinic receptors, contributing to its notably favorable tolerance profile [13]. Its extensive receptor profile, combined with its capability to indirectly modulate the glutamate pathway and its relatively limited adverse effects compared to other atypical antipsychotics, suggest that lumateperone could be effectively used for the rapid treatment of schizophrenia and depression (at higher doses) as well as for disorders accompanied by agitation, aggression, or sleep disturbances (at lower doses) [14].

### 2.2. Pharmacokinetics

In vitro studies demonstrate that lumateperone exhibits high bi-directional permeability through the Caco-2 cell monolayer, an immortalized human colorectal adenocarcinoma cell line used as a model of the intestinal epithelial barrier. These studies indicate that lumateperone penetrates cells primarily through passive diffusion, suggesting that the drug should have high intestinal absorption with low efflux potential [3]. Additionally, it has been shown that lumateperone can cross the blood–brain barrier, primarily due to its significant multidrug resistance protein 1 (MDR1) permeability. The compound also binds significantly to cytochrome P450 3A4 (CYP3A4) in the ileum, a property attributed to its high lipophilicity at physiological pH (pH 7.4) [15].

#### 2.2.1. Absorption

Peak plasma concentration (C_max_) of lumateperone is reached approximately 1 to 2 h after oral administration, with an absolute bioavailability of 4.4%. The compound, at concentrations ranging from 0.05 to 50 ng/mL, and its metabolites, at concentrations ranging from 0.2 to 100 ng/mL, can be detected in the plasma for up to 8 h [12,16]. Notably, consuming high-fat meals with lumateperone results in approximately a 33% decrease in C_max_, a 9% increase in AUC_inf_, and a delay in T_max_ by about one hour [3]. Clinical trials have shown a higher incidence of gastrointestinal (GI) effects in the higher-dose groups; hence, the reduced C_max_, when taken with food, may lead to fewer such effects. Therefore, it is recommended to take lumateperone with food [3].

Steady-state concentrations, which increase approximately dose-proportionately over a dosage range from 21 mg to 56 mg, are achieved after approximately five days of once-daily oral administration. There is considerable inter-individual variability in the pharmacokinetic parameters of lumateperone, with coefficients of variation for C_max_ and AUC ranging from 68% to 97% for the therapeutic dose of 42 mg at steady state [3].

This significant inter-patient variability underscores the need for individualized considerations in clinical practice. Factors such as metabolic enzyme activity, concomitant medications, and genetic polymorphisms may play a role in these differences, potentially influencing both efficacy and tolerability. Expanding the understanding of pharmacokinetic diversity in diverse patient populations is crucial for optimizing dosing strategies and improving therapeutic outcomes.

#### 2.2.2. Distribution

Human plasma protein binding of lumateperone is 97.4% at a concentration of 5 μM, approximately 70-fold higher than the therapeutic concentration, while the volume of distribution following intravenous administration is about 4.1 L/kg [3,15]. After the oral administration of labelled (^14^C) lumateperone at 7 mg/kg, peak levels of radioactivity in rat’s blood and tissue (except the meninges) were reached 6 h post-dosing. The highest levels of radioactivity were detected in the gastrointestinal (GI) tract, liver, bladder, adrenal glands, kidneys, iron, eyes (choroid, retina), and pancreas. Radioactivity remained higher in most tissue than in whole blood throughout the study period, extending up to 840 h. Significant radioactivity was also observed in the brain and spinal cord [3]. Lumateperone was rapidly distributed to the brain in less than 1 h. The high brain-to-plasma ratio (1:3.4 to 1:21) is likely due to the lack of affinity for the human efflux transporter BCRP [3].

Following oral administration of lumateperone at a 7 mg/kg dose, two pharmacologically active metabolites, IC200161 and IC200131, were also detectable in the brain. The first metabolite was found at 1 h and 4 h post-administration, but its levels were approximately 10-fold lower than those of lumateperone. The second metabolite, IC200131, was at the limit of quantification. These findings suggest that the primary pharmacological effect depends on lumateperone itself [3].

#### 2.2.3. Metabolism

After oral administration, lumateperone undergoes extensive metabolism in both humans and non-clinical species (such as rats), leading to the formation of over 20 different metabolites. Importantly, significant interspecies differences in metabolic pathways have been observed. In humans, the primary metabolic pathway involves a reduction in the ketone in the butyrophenone side chain, forming the reduced carbonyl metabolite IC200131. In contrast, in non-clinical species, the predominant pathway is the demethylation of the piperazine ring, resulting in the N-desmethyl metabolite IC200161. Additionally, in humans, glucuronidation of the butyrophenone side chain at the enol/keto position predominates in phase II metabolism, which appears less important in non-clinical species.

In human hepatocytes, primary phase I metabolic pathways, aside from the reduction in the ketone mentioned above to form IC200131 (24.78%), include the generation of the secondary N-desmethyl metabolite IC200565 (10.41%) and amidation leading to the tertiary metabolite IC201309 (6.39%) [3,15,17]. The conversion of lumateperone to IC200131 is primarily mediated by enzymes from the aldo-keto reductase (AKR) family, particularly AKR1C1, and to a lesser extent, AKR1B10, while AKR1C4 and AKR1C3 play minimal or no role.

Several cytochrome P450 enzymes (CYP3A4, CYP2C8, CYP1A2, CYP2A6) are involved in phase I reactions. Specifically, CYP3A4 metabolizes IC200131 to IC200565 (N-demethylated alcohol metabolite) or back to lumateperone. Phase II metabolism involves the glucuronidation of lumateperone and its metabolites through several enzymes from the uridine diphosphoglucuronosyl transferase (UGT) family, suggesting that glucuronidation may be the major metabolic pathway in humans. Key enzymes include UGT1A1, UGT1A4, and UGT2B15 for lumateperone; UGT2B7, UGT2B15, and UGT2B17 for IC200131; UGT2B10 and UGT2B15 for IC200161; UGT1A3 for IC200565; and UGT1A1, UGT1A3, UGT1A4, UGT1A9, UGT2B7, and UGT2B17 for IC201309 [3,15,17].

#### 2.2.4. Excretion

The half-life (T_1/2_) of lumateperone ranges from 13 to 21 h following oral administration and is approximately 18 h after intravenous administration. The half-life of its primary metabolites is 20 h for ICI200161 and 21 h for ICI200131 [12]. Due to the size of the lumateperone molecule, only a negligible amount (less than 1% of the dose) is excreted unchanged in the urine; lumateperone is primarily excreted in the feces. In a human mass balance study, 58% of the radioactive dose was recovered in the urine and 29% in the feces. The clearance of lumateperone is approximately 27.9 L/h. Its metabolites are highly soluble in water and, as such, are entirely excreted in humans [3,15,17,18].

#### 2.2.5. Drug–Drug Interactions

Given that lumateperone is metabolized by CYP3A4 and UGT enzymes, drug interactions are possible. Consequently, concomitant use with CYP3A4 inducers (e.g., carbamazepine) and inhibitors (e.g., ketoconazole, fluvoxamine) should be avoided. Similarly, it is not recommended to use lumateperone in combination with UGT inhibitors (e.g., valproic acid) [3,15]. Additionally, the drug may interact with alcohol and sedatives [19].

### 2.3. Pharmacodynamics: Receptor-Binding Profile

The mechanism of action of lumateperone involves complex modulation of serotonergic, dopaminergic, and glutamatergic neurotransmission, mediated by a unique combination of receptor/transporter-binding affinities [4]. Radioligand displacement and functional profile studies have indicated that lumateperone exhibits high-affinity binding to 5-HT_2A_ receptors (K_i_ = 0.54 nM) and inhibits serotonin-induced (30 nM) increases in calcium fluorescence with an IC_50_ of 7 nM [20]. Additionally, lumateperone demonstrates moderate affinity for D_2_ receptors (K_i_ = 32 nM; selectively in the mesolimbic and mesocortical regions), D_1_ receptors (K_i_ = 52 nM), and the SERT (K_i_ = 62 nM). Conversely, it shows low affinity for α_1_ adrenergic receptors (K_i_ = 73 nM), 5-HT_2C_ serotonin subtype receptors (K_i_ = 173 nM), and H_1_ histamine receptors (K_i_ > 1000 nM) [8,18,20].

In two published positron emission tomography (PET) studies, lumateperone (taken orally in doses of 10, 40, and 60 mg) was found to be safe and well tolerated, and rapidly entered the human brain, with dose-related occupancy rates of 5-HT_2A_ and D_2_ receptors [10,16].

#### 2.3.1. Serotonergic Neurotransmission: 5-HT_2A_ Receptors and SERT

Lumateperone is a potent antagonist of the 5-HT_2A_ receptor and an inhibitor of the SERT [12]. Preclinical (animal) studies suggest that at low doses (<0.2 mg/kg), lumateperone acts as a selective 5-HT_2A_ receptor antagonist [20]. In a positron emission tomography (PET) study involving healthy volunteers (N = 16), a single oral dose of 10 mg lumateperone demonstrated high occupancy of cortical 5-HT_2A_ receptors in the brain, with occupancy rates exceeding 80%. At a higher dose (40 mg), the drug exhibited occupancy of striatal serotonin transporters ranging from 8% to 33% [16]. Notably, the affinity of lumateperone for SERT is more potent than that of other antipsychotics, such as olanzapine (K_i_ > 1000) and aripiprazole (K_i_ 240–405) [8].

#### 2.3.2. Dual Modulation of D_2_ Receptors

Depending on their synaptic localization, lumateperone has a dual function in modulating D_2_ receptors. Unlike haloperidol (a first-generation antipsychotic) or risperidone (a second-generation antipsychotic, SGA), lumateperone acts as a presynaptic agonist and a postsynaptic antagonist at D_2_ receptors [21]. This dual modulation contributes to its ability to reduce psychotic symptoms while minimizing the risk of extrapyramidal symptoms (EPSs) and other dopamine-related side effects.

The mechanism by which lumateperone functions as a postsynaptic D_2_ receptor antagonist may involve a dose-dependent increase in glycogen synthase kinase 3 (GSK-3) phosphorylation at serine 9 (S9), primarily observed in the prefrontal cortex and nucleus accumbens of mice [20]. This functional selectivity at D_2_ receptors in the mesolimbic and mesocortical regions contrasts with its lower affinity for the nigrostriatal pathway, which likely underpins its favorable safety profile [12,22].

In a positron emission tomography (PET) clinical study involving healthy volunteers (N = 16), a single oral dose of 10 mg lumateperone induced low occupancy of striatal D_2_ receptors (D_2_ receptor occupancy, D2RO ~12%). D2RO was dose- and plasma concentration-dependent, with a higher dose (40 mg, single oral dose) increasing striatal D2RO by up to 39% [16]. Moreover, in a PET study involving patients with schizophrenia (N = 10) who received 60 mg lumateperone orally once daily for 14 days, a mean peak dorsal striatal D2 receptor occupancy of 39% was observed one hour post-dose [10]. These occupancy levels are notably lower than those observed with risperidone (4 mg/day; D2RO = 72–81%) or olanzapine (5–60 mg/day; D2RO = 61–80%) [8,10].

This lower D2RO suggests that lumateperone exerts its therapeutic effects while maintaining a reduced risk of motor side effects commonly associated with higher D_2_ receptor occupancy. Additionally, the dual role of lumateperone in modulating presynaptic and postsynaptic D_2_ receptors highlights its potential to address both positive and negative symptoms of schizophrenia, as well as cognitive impairments, through region-specific dopaminergic regulation.

#### 2.3.3. Dopaminergic and Glutamatergic Neurotransmission: D_1_-Dependent Regulation of NMDA Receptors

Lumateperone has demonstrated the ability to modulate glutamatergic neurotransmission [11]. The primary proposed molecular mechanism behind this action is thought to be associated with D_1_ receptor-dependent enhancement of phosphorylation of the GluN2B subunit of N-methyl-D-aspartate (NMDA) receptors [23,24,25]. For instance, Snyder et al. observed that lumateperone (3 mg/kg, administered orally) increased the phosphorylation level at tyrosine (Y) 1472 of the GluN2B subunit in the nucleus accumbens of mice [20]. Additionally, an increased level of α-amino-3-hydroxy-5-methyl-4-isoxazolepropionic acid (AMPA) receptors, mediated through the mammalian target of rapamycin (mTOR) pathway, has been described [22]. However, the pharmacological mechanisms underlying lumateperone’s modulation of glutamatergic signaling, encompassing both AMPA and NMDA receptors, require further investigation.

#### 2.3.4. Modulation of NMDA/AMPA Signaling and the mTOR Pathway

Lumateperone enhances glutamatergic neurotransmission through modulation of NMDA and AMPA receptors. Specifically, it increases phosphorylation of the GluN2B subunit of NMDA receptors, enhancing receptor function [8,20]. Additionally, lumateperone promotes AMPA receptor trafficking and function by activating the mTOR signaling pathway [26]. This modulation of glutamate receptors may improve cognitive function and negative symptoms.

### 2.4. Antipsychotic-like and Antidepressant-like Activity of Lumateperone: Animal (Preclinical) Studies

Lumateperone is a pharmacodynamically novel, first-in-class antipsychotic that modulates serotonin, dopamine, and glutamate neurotransmission [4,20]. Preclinical studies suggest that the drug may exhibit antipsychotic-like activities and improve upon antidepressant-like efficacy [20]. Evidence indicates that lumateperone (0.09 mg/kg, administered orally) effectively blocked head-twitch behaviors in mice induced by 2,5-dimethoxy-4-iodoamphetamine hydrochloride (DOI; 2.5 mg/kg, intraperitoneal; a 5-HT_2_ agonist). Additionally, the drug inhibited amphetamine-induced (D-AMPH; 1 mg/kg, intraperitoneal) hyperactivity in rats with an ID50 of 0.95 mg/kg (administered orally). Notably, the results for lumateperone were comparable to those observed with risperidone, which showed an ID50 of 0.33 mg/kg (administered orally) in the D-AMPH test. Furthermore, single oral administrations of lumateperone (1–10 mg/kg) had no significant effect on motor performance in mice, as assessed by forelimb catalepsy [20].

These in vivo studies confirm the potential antipsychotic-like activity of lumateperone, primarily mediated through 5-HT_2A_ and postsynaptic D_2_ receptor antagonism. On the other hand, in a chronic social defeat stress (CSDS) model of depression, 28-day administration of lumateperone (1 mg/kg, intraperitoneal) led to only a non-significant reduction in social behavior in mice [20]. It should be noted that chronic (28-day) oral administration of lumateperone at a dose of 3.2 mg/kg/day resulted in a significant reduction in total activity time in the forced swim test (FST) and fewer crossings in the open field test (OFT) in guinea pigs, along with histopathological deterioration in adrenal secretory function [27]. These results are consistent with the FDA Caplyta approval report, which lists possible treatment side effects of lumateperone, such as hypoactivity and splayed posture [3,27].

Interestingly, the co-administration of quercetin (50 mg/kg/day), an antioxidant and anti-inflammatory agent, with lumateperone (3.2 mg/kg/day) for 28 days increased total activity time and decreased immobilization time in the FST. These changes in general locomotor activity suggest potential antidepressant-like effects from the combined treatment with quercetin and lumateperone. Moreover, quercetin may prevent the side effects associated with lumateperone [27]. The mechanisms underlying the therapeutic actions of combined quercetin (or other antioxidant/anti-inflammatory agents) and lumateperone treatments need to be further investigated in future studies. The antidepressant activity of lumateperone is also confirmed by studies indicating a dose-dependent reduction in abnormally elevated levels of key pro-inflammatory cytokines (e.g., IL-1β, IL-6, and TNF-α) both in the brain and in the serum of mice in response to an inflammatory challenge with using a single dose of LPS (500 μg/kg). Moreover, lumateperone also reduced the levels of pro-inflammatory cytokines in male rats subjected to acute behavioral stress while exhibiting anxiolytic-like and antianhedonic-like effects. These changes were accompanied by increased activity of the mammalian target of rapamycin complex 1 pathway in the prefrontal cortex [26].

### 2.5. Role of Lumateperone Pharmacodynamics Properties in Clinical Efficacy

In general, lumateperone demonstrates several unique and significant dose-related interactions with 5-HT_2A_, D_2_, D_1_, and NMDA receptors and the SERT [16]. The clinical implications of lumateperone’s action on 5-HT_2A_ and D_2_ receptors are relevant for a reduction in extrapyramidal side effects (EPS), which are typical of commonly used pharmacotherapies for schizophrenia [28]. Lumateperone exhibits a higher binding affinity for 5-HT_2A_ receptors than D_2_ receptors, with a ratio of 1:60 [29]. Hypothetically, lumateperone should lead to increased binding to 5-HT_2A_ receptors without over-activating D_2_ receptors, thereby preventing possible motor side effects [14,29].

Results from PET clinical studies in schizophrenia patients have shown lower dorsal striatal D_2_ receptor occupancy after lumateperone (60 mg) treatment compared with other antipsychotic drugs [10]. As a presynaptic D_2_ partial agonist, lumateperone modulates presynaptic D_2_ activity without complete antagonism [29]. This may explain its favorable safety and efficacy profile. Importantly, lumateperone is regioselective for D_2_ receptor activity in the mesolimbic and mesocortical systems, which could promote more effective antipsychotic activity for both the negative and positive symptoms of schizophrenia [12]. Simultaneously, its low binding affinity to α_1_ adrenergic, H_1_ histaminergic, and 5-HT_2C_ serotonergic receptors contributes to a favorable tolerability profile, minimizing the risk of metabolic and cardiovascular complications [30].

Lumateperone’s therapeutic efficacy is also associated with its moderate binding affinity to the SERT. It is suggested that the modulation of serotonergic neurotransmission through synergistic 5-HT_2A_ antagonism and SERT inhibition can enhance the potential antidepressant effects of lumateperone [12,31]. The antidepressant activity of this drug may also be related to the regulation of NMDA receptor function [28].

It should be noted that lumateperone exhibits different therapeutic effects depending on the dosage. At relatively low doses, it acts selectively as a 5-HT_2A_ antagonist, which can produce anti-aggressive effects. At higher doses, primarily due to its ability to optimize dopamine levels, lumateperone may reduce depressive and psychotic symptoms [12,29,32].

## 3. Clinical Considerations

This article began with an examination of the pharmacological properties of lumateperone. This drug has a distinctive mechanism of action characterized by the modulation of serotonergic, dopaminergic, and glutamatergic neurotransmission, setting it apart from other currently available antipsychotic medications [13]. The FDA approved lumateperone for the treatment of schizophrenia in adults based on the results of a Phase 3 double-blind, placebo-controlled clinical trial [19].

The second part of this article will focus on the clinical aspects of lumateperone use. This will include a discussion of recent clinical trials investigating its efficacy and safety across various patient populations, not limited to those with schizophrenia [4]. Additionally, it will present comparisons with other antipsychotics, delve into lumateperone’s mechanism of action, and explore its use in different patient groups.

### 3.1. Issues of Efficacy and Effectiveness

While there are relatively few clinical trials on lumateperone, making it challenging to comprehensively compare its effectiveness with other antipsychotic drugs [28,33,34], some general conclusions can nevertheless be drawn.

According to Stahl [35], although lumateperone was approved by the FDA for the treatment of schizophrenia and bipolar depression (as monotherapy or add-on therapy for the latter), its range of potential indications is much broader. The spectrum of indications extends from acute mania [36,37,38] to post-traumatic stress disorder (Table 1).

The timeframe for treatment with lumateperone does not significantly differ from that of other antipsychotics. While it takes approximately one week to observe meaningful improvement in psychotic or manic symptoms, the full range of therapeutic effects—encompassing affective, cognitive, and behavioral dimensions—develops over several weeks [39]. The decision on whether to continue with lumateperone or switch to another D_2_ antagonist in patients with schizophrenia is typically made after 4–6 weeks of treatment. Notably, if the initial effects are promising, it may take 4–5 months to observe comprehensive improvement in the patient’s mental status, particularly concerning cognitive and negative symptoms. In quantitative terms, it is reasonable to expect an overall symptomatic improvement of about 33% in patients with schizophrenia and around 50% in cases of mania, excluding individuals suffering from treatment resistance [9].

### 3.2. Side Effects

The most common adverse events associated with lumateperone treatment are sedation, dry mouth, and nausea. While the evidence on the following is less certain, vigilance is necessary for the onset of non-specific antipsychotic-related side effects such as drug-induced parkinsonism, tardive dyskinesia, hyperglycemia, and other metabolic disturbances, as well as rare occurrences of seizures and neuroleptic malignant syndrome. It is noteworthy that the risk of movement disorders tends to increase with the duration of treatment and cumulative dose of the medication. However, the likelihood of weight gain with lumateperone therapy is insignificant [40,41].

Given that sedation can impair daytime activities for patients taking lumateperone, administering the drug right before bedtime might mitigate this issue [9].

### 3.3. Other Practical Remarks

Lumateperone is taken at a dose of 42 mg daily, as a single daily dose, with no need for titration. Given this fixed dosing regimen, alternative strategies must be considered in certain challenging clinical scenarios. For instance, in acutely agitated patients, combining lumateperone with a benzodiazepine or another antipsychotic is recommended. In patients with schizophrenia who exhibit only a partial response to lumateperone, adding a mood-stabilizing agent like lamotrigine or topiramate may be beneficial [9].

Although the rationale is unclear given the drug’s relatively stable pharmacokinetics irrespective of the patient’s satiety status, it is formally advised that lumateperone be taken with food. This recommendation arises from the drug being tested exclusively under such conditions [9].

A decrease in the absolute neutrophil count to below 1000/mm^3^ necessitates the urgent discontinuation of lumateperone treatment [9].

Unfortunately, there is scant evidence regarding the effectiveness and safety profile of lumateperone in special populations such as the elderly, pregnant women, and individuals with renal, cardiac, hepatic, or other medical issues [9,42,43].

Overall, lumateperone appears to be a promising treatment option for patients who cannot tolerate other antipsychotics, those requiring a rapid onset of therapeutic effects without the need for dose titration, and individuals at high risk of developing movement disorders or metabolic complications.

In summary, lumateperone addresses not only the positive and negative symptoms of psychosis but also cognitive impairments and affective disturbances associated with bipolar disorder, including both manic and depressive episodes. Furthermore, it has shown potential in managing aggression. Preliminary evidence also suggests that lumateperone may improve functional outcomes in patients with schizophrenia, particularly in enhancing social interactions [9].

### 3.4. Comparative Efficacy and Safety

Lumateperone’s unique pharmacological profile sets it apart from traditional SGAs such as risperidone and olanzapine. Risperidone has high affinity for D_2_ and 5-HT_2A_ receptors but is associated with a higher risk of extrapyramidal symptoms (EPSs) and hyperprolactinemia due to strong D_2_ receptor antagonism. Olanzapine effectively reduces psychotic symptoms but is often linked to significant weight gain and metabolic disturbances because of its affinity for H_1_ histaminergic and 5-HT_2C_ receptors [41,44,45].

In contrast, lumateperone demonstrates moderate affinity for D_2_ receptors and high affinity for 5-HT_2A_ receptors, with minimal activity at H_1_ and 5-HT_2C_ receptors. Clinical trials have shown that lumateperone is effective in reducing both positive and negative symptoms of schizophrenia, with a lower incidence of EPSs and metabolic side effects compared to risperidone and olanzapine [4,18,40]. Moreover, lumateperone’s modulation of glutamatergic neurotransmission may contribute to cognitive improvements not typically seen with other SGAs [2,8].

A summary of the notable clinical trials on lumateperone is provided in Table 2.

### 3.5. Potential Novel Indications

Recent research has explored the use of lumateperone in conditions beyond schizophrenia and bipolar disorder. Preclinical studies suggest that lumateperone may have therapeutic potential in treating post-traumatic stress disorder (PTSD), anxiety disorders, and neurodegenerative disorders [26,46].

In animal models of PTSD, lumateperone has been shown to reduce anxiety-like behaviors and modulate stress-induced neurochemical changes [45]. Its effects on glutamatergic neurotransmission and the mTOR pathway may underlie its potential benefits in neurodegenerative disorders, where dysregulation contributes to disease progression [26,47,48,49]. Ongoing clinical trials are investigating lumateperone’s efficacy in these novel indications, highlighting its potential as a versatile therapeutic agent.

## 4. Discussion

Lumateperone offers a distinctive approach to neurotransmission, engaging serotoninergic, dopaminergic, and glutamatergic systems. This multifaceted mechanism sets it apart from conventional antipsychotics, which primarily target dopaminergic pathways and are often associated with a higher prevalence of extrapyramidal symptoms (EPSs) [1,2]. Clinical trials have demonstrated the efficacy of lumateperone in reducing both positive and negative symptoms of schizophrenia, as well as its potential utility in the treatment of bipolar depression [37,41].

Furthermore, the potential of lumateperone extends beyond its immediate therapeutic applications, with emerging research suggesting that it may play a significant role in addressing treatment-resistant forms of mood disorders and behavioral symptoms associated with neurodegenerative diseases. Studies indicate that lumateperone’s distinctive mechanism, acting as both an antagonist at 5-HT_2A_ receptors and a modulator of dopaminergic activity, could offer a distinct advantage in managing complex cases where traditional antipsychotics are ineffective [2,9]. Furthermore, its favorable safety profile, particularly the reduced risk of EPS compared to other agents, makes it a compelling option for vulnerable populations, such as the elderly or those with pre-existing movement disorders. The capacity of lumateperone to selectively modulate these neurotransmitters may confer a therapeutic advantage, particularly in patients susceptible to the adverse effects of conventional treatments.

One of the most compelling aspects of lumateperone is its favorable side-effect profile. In contrast to numerous antipsychotic medications that have been associated with substantial weight gain, metabolic syndrome, and movement disorders, lumateperone exhibits a reduced propensity for these adverse effects [40,41]. This is of particular importance concerning long-term treatment adherence, as patients are often reluctant to continue taking medications that induce uncomfortable or harmful side effects. Although the recommendation to administer lumateperone with food may appear to be at odds with its pharmacokinetic stability, it may help mitigate gastrointestinal disturbances, further enhancing patient compliance [35]. Nevertheless, this assumption requires verification in future clinical trials.

The pharmacokinetic characteristics of lumateperone, including its half-life and metabolic pathways, indicate that a manageable dosing regimen may be achievable. Without titration, the fixed daily dose of 42 mg simplifies treatment protocols, which can be particularly beneficial in acute settings or among populations with limited access to regular medical supervision. Although the potential for drug–drug interactions, particularly with CYP3A4 and UGT inhibitors, necessitates careful monitoring, the overall pharmacodynamic profile of lumateperone suggests a promising avenue for the development of personalized treatment strategies [10,50].

Despite the encouraging preliminary findings, further investigation is required to address several outstanding issues. The long-term safety profile of lumateperone remains to be fully elucidated, particularly in vulnerable populations such as the elderly, pregnant women, and individuals with pre-existing health conditions [9,42,43]. Additionally, while preliminary evidence suggests potential efficacy in a broader range of indications, including post-traumatic stress disorder and behavioral disorders in children, these hypotheses require rigorous clinical validation.

In conclusion, lumateperone represents a significant advancement in antipsychotic therapy, offering a distinctive pharmacological profile and the potential for an optimal balance of safety and efficacy. Ongoing and future studies must be conducted to establish the role of lumateperone in the treatment of schizophrenia and other complex disorders, with the goal of improving the quality of life for patients affected by these disorders. As the clinical landscape continues to evolve, integrating lumateperone into treatment paradigms may offer new hope for those who have struggled with the limitations of existing therapies [9].

## Figures and Tables

**Table 1:** Preliminary list of potential indications for treatment with lumateperone.

Schizophrenia and other primary psychiatric disorders Bipolar depression (either monotherapy or combination treatment) Acute, “clean” mania or mania with mixed features Maintenance treatment of BD Treatment-resistant MDD Behavioral disorders in the course of dementia Behavioral disorders in children and adolescents Impulse-control disorders PTSD

BD—bipolar disorder, MDD—major depressive disorder, PTSD—post-traumatic stress disorder. Adapted from Stahl [9].

**Table 2:** Summary of key clinical trials on lumateperone.

Study	Patient Population	Study Design	Dose(s) of Lumateperone	Efficacy Outcomes	Safety Profile
Correll et al., 2020 [19]	N = 450 patients with schizophrenia	Phase 3, randomized, double-blind, placebo-controlled	42 mg/day	Significant reduction in PANSS total score vs. placebo	Similar incidence of adverse events as placebo; low rates of EPS
Calabrese et al., 2021 [36]	N = 381 patients with bipolar depression	Phase 3, randomized, double-blind, placebo-controlled	42 mg/day (monotherapy)	Significant improvement in MADRS score vs. placebo	Well tolerated; minimal weight gain
Suppes et al., 2022 [38]	N = 554 patients with bipolar depression	Phase 3, adjunctive therapy with lithium or valproate	42 mg/day	Greater reduction in depressive symptoms vs. adjunctive placebo	Favorable safety profile; low risk of metabolic side effects

MADRS—Montgomery–Åsberg Depression Rating Scale; PANSS—Positive and Negative Syndrome Scale.

## Data Availability

This review article relies exclusively on previously published data, fully cited in the bibliography. The original datasets used in the cited studies were not accessed or analyzed by the authors.
